# The Regulatory Effects of Long Noncoding RNA-*ANCR* on Dental Tissue-Derived Stem Cells

**DOI:** 10.1155/2016/3146805

**Published:** 2016-08-28

**Authors:** Qian Jia, Xiaolin Chen, Wenkai Jiang, Wei Wang, Bin Guo, Longxing Ni

**Affiliations:** ^1^Institution of Stomatology, The PLA General Hospital, No. 28 Fuxing Road, Beijing 100853, China; ^2^Department of Endodontics, Xiamen Stomatology Hospital, Teaching Hospital of Fujian Medical University, No. 1309 Lvling Road, Xiamen, Fujian 361003, China; ^3^State Key Laboratory of Military Stomatology & National Clinical Research Center for Oral Diseases & Shaanxi Key Laboratory of Oral Diseases, Department of Operative Dentistry & Endodontics, School of Stomatology, Fourth Military Medical University, No. 145 Western Changle Road, Xi'an, Shaanxi 710032, China

## Abstract

Long noncoding RNAs (lncRNA) have been recognized as important regulators in diverse biological processes, such as transcriptional regulation, stem cell proliferation, and differentiation. Previous study has demonstrated that lncRNA-*ANCR* (antidifferentiation ncRNA) plays a key role in regulating the proliferation and osteogenic differentiation of periodontal ligament stem cells (PDLSCs). However, little is known about the role of* ANCR* in regulating other types of dental tissue-derived stem cells (DTSCs) behaviours (including proliferation and multiple-potential of differentiation). In this study, we investigated the regulatory effects of lncRNA-*ANCR* on the proliferation and differentiation (including osteogenic, adipogenic, and neurogenic differentiation) of DTSCs, including dental pulp stem cells (DPSCs), PDLSCs, and stem cells from the apical papilla (SCAP) by downregulation of lncRNA-*ANCR*. We found that downregulation of* ANCR* exerted little effect on proliferation of DPSCs and SCAP but promoted the osteogenic, adipogenic, and neurogenic differentiation of DTSCs. These data provide an insight into the regulatory effects of long noncoding RNA-*ANCR* on DTSCs and indicate that* ANCR* is a very important regulatory factor in stem cell differentiation.

## 1. Introduction

Dental tissue-derived stem cells (DTSCs) are stem cells separated from the dental tissue that have self-proliferation and multidirectional differentiation potential. Dental pulp stem cells (DPSCs), periodontal ligament stem cells (PDLSCs), and stem cells from the apical papilla (SCAP) are three important DTSCs which are used in tissue engineering research [1–4]. Hilkens et al. found that DPSCs are capable of osteogenic, adipogenic, and chondrogenic differentiation via different induction methods [5]. Kim et al. induced differentiation of DPSCs into cementoblast and neurocytes-like cells [6]. Dissanayaka and Janebodin found that DPSCs could be differentiated into vascular endothelial cells and had angiopoietic abilities after stimulation with an angiopoietic factor [7, 8]. Seo et al. combined PDLSCs with HA/TCP and implanted the complex into a nude mouse where the PDLSCs formed periodontium and cementum-like tissues [2]. Sonoyama et al. seeded PDLSCs and SCAP mixed cells into HA/TCP and implanted the complex into young swine where the mixed cells formed functional bioroot and periodontium [9].

Many factors, including growth factors, inflammatory factors, and microRNAs, play important roles in regulating the proliferation and differentiation of DTSCs (Table 1) [7, 10–25]. After lucubration on the regulatory effects of microRNAs on DTSCs, scholars discovered that many noncoding RNAs were key regulating factors in the proliferation and differentiation of DTSCs [14–16].

While 93% of human genomic DNA is translated into RNA, only 2% of RNA is transcribed into protein. Most of the DNA is translated into noncoding RNAs [26]. Long noncoding RNA (lncRNA) used to be recognized as useless transcriptional noise [27]. In recent years, scientists discovered that they were very important in regulating epigenetic, transcriptional, and posttranscriptional gene expression [26–31]. Antidifferentiation noncoding RNA (*ANCR*) is a newly found lncRNA that is downregulated during stem cell differentiation and required to keep epidermal stem cells or osteoblast cells in an undifferentiated cell state. Depleting* ANCR* in progenitor-containing populations, without any other stimuli, resulted in rapid differentiation and gene induction [32]. Zhu and Xu found that downregulating* ANCR* promoted osteoblast differentiation by targeting* EZH2* and regulating* Runx2* expression [33]. Additionally, our previous research also showed that* ANCR* promoted the osteogenic differentiation of PDLSCs [34]. In this experiment, we used RNA interference to downregulate* ANCR* expression to further analyze the regulatory function of* ANCR* on the proliferation and differentiation of DTSCs, which would give a laterally comparative view on the regulatory function of* ANCR* on DTSCs.

## 2. Materials and Methods

### 2.1. Sample Collection and Cell Culture

Healthy impacted third molars were collected from adult humans between 19 and 29 years old from the dental hospital of the Fourth Military Medical University. All tooth extractions were conducted under the approval of the Ethical Committee of School of Stomatology, Fourth Military Medical University (permission number IRB-REV-2014-018). Informed consent was obtained from all subjects and the methods were carried out in accordance with the approved guidelines. Tissues from the dental pulp, periodontal ligament, and apical papilla were isolated from each molar, as previously described [1–3, 35]. Briefly, the tissues were gently separated and digested in a solution composed of 3 mg/mL of collagenase type I (Invitrogen, USA) and 4 mg/mL of dispase (Invitrogen, USA) for 40 min at 37°C. The cell suspension was obtained by passing the solution through a 70-*μ*m strainer. Then, 1 × 10^4^ cells were seeded into each well of a 6-well plate. *α*-modification of Eagle's Medium (*α*-MEM, Invitrogen, USA) supplemented with 10% fetal bovine serum (HyClone, USA), 100 mol/L of L-ascorbic acid 2-phosphate (Sigma, USA), 2 mmol/L of L-glutamine (Sigma, USA), 100 U/mL of penicillin, and 100 *μ*g/mL of streptomycin (Gibco, USA) was used as the standard culturing medium for cells at 37°C with 5% CO_2_. The medium was changed every 2-3 days. A limited dilution technique was applied and single cells were seeded into a 96-well plate to obtain single cell–derived colonies. Upon reaching 80% confluency, a number of these single-colony-derived lines were passaged at a 1 : 3 ratio for further culturing.

### 2.2. Immunophenotype Analysis

DTSCs were stained with stem cell surface markers and analyzed by flow cytometry as described previously [16]. Briefly, to identify the phenotypes of DTSCs, 5 × 10^5^ cells at the 3rd passage were incubated with phycoerythrin (PE) conjugated monoclonal antibodies for human* CD29, CD34, CD45, CD90,* and* CD146* or Allophycocyanin (APC) conjugated monoclonal antibody against human* STRO-1*, based on the manufacturer's instructions. The incubation procedure was carried out at 4°C away from light for 1 h. After washing with PBS, the cells were subjected to flow cytometric analysis.

### 2.3. Lentivirus Infection

The third-passage self-inactivating lentivirus vector was purchased from Neuron Biotech (Shanghai Neuron Biotech Co., Ltd., Shanghai, China). The vector contained a puromycin marker and U6 PolIII promoter, which allowed the introduction of oligonucleotides encoding short hairpin RNAs (shRNAs) and synthetic oligonucleotides containing the human* ANCR* (NCBI accession number NR_024031.1) splice variant target sequence (GCTGACCCTTACCCTGAATAC). The sequences for cloning were synthesized, annealed, and ligated into the pLKD-CMV-G lentiviral vector between the AgeI and EcoRI enzyme sites after the U6 promoter. The oligonucleotide sequences were 5′-CCGGGCTGACCCTTACCCTGAATACCTCGAGGTATTCAGGGTAAGGGTCAGCTTTTTT-3′ (sense); and 5′- AATTCAAAAAAGCTGACCCTTACCCTGAATACCTCGAGGTATTCAGGGTAAGGGTCAGC-3′ (antisense). DTSCs at passage 3 were plated at a density of 5 × 10^4^ cells/well into 6-well plates. The cells were cultured for 3 days along with recombinant lentivirus encoding shRNA against* ANCR* at a multiplicity of infection (MOI) of 10, in *α*-MEM supplemented with 10% FBS containing 5 mg/mL of polybrene at 37°C and 5% CO_2_. The cells were then replated in 25-cm^2^ flasks in 90%  *α*-MEM, 10% FBS, and 5 *μ*g/mL of puromycin and cultured at 37°C with 5% CO_2_ and constant humidity. The* ANCR* expression after* ANCR*-RNA interference (RNAi) was examined by qRT-PCR analysis. Cells successfully infected with specific* ANCR*-RNAi were designated as DPSC/*ANCR*-RNAi, PDLSC/*ANCR*-RNAi, and SCAP/*ANCR*-RNAi, while the wild-type DTSCs were designated as DPSC/wt, PDLSC/wt, and SCAP/wt and cells infected by control lentivirus vector were designated as DPSC/vector, PDLSC/vector, and SCAP/vector.

### 2.4. Cell Viability Assay (CCK8 Kit)

Cells were seeded into two 96-well plates at a density of approximately 1 × 10^4^ cells per well. The assay was performed 24 h after seeding and lasted for 7 days; data were collected at the same time point each day. A Cell Counting Kit-8 (CCK8) was used to perform this experiment. The number of cells per well was tested by the absorbance (450 nm) of reduced WST-8 at the indicated time points.

### 2.5. Cell Viability Assay (Edu Kit)

Cells were seeded into two 96-well plates at a density of approximately 1 × 10^4^ cells per well and cultivated to logarithmic phase. Then, the cell viability assay was performed with Edu kit (RiboBio, China) as the manufacturers' instruction. Briefly, 50 *μ*M 5-ethynyl-2′-deoxyuridine (Edu) was added into the culture medium and cells were cultured for 2 hours. After 2-time PBS washing and paraformaldehyde fixation, the cell nucleus were stained with Apollo 567 and Hoechst 33342. Then, the cells were imaged with a laser scanning confocal microscope (Zeiss, Germany) at 550 nm and 350 nm in the same vision. Then, the images of the same vision were overlapped in one image. Cell counting was done by Image J.

### 2.6. Alkaline Phosphatase (ALP) Activity Quantification

Cells were seeded into 96-well plates at approximately 1 × 10^5^ cells per well. 24 h after seeding, the medium was changed into standard osteogenic differentiation induction medium (50 mg/mL of ascorbic acid (Sigma, USA)), 10 mmol/L of beta-glycerophosphate (BGP, Sigma, USA) and 10 ng/mL of dexamethasone diluted in 10% FBS *α*-MEM.* ALP* quantification assay was performed at 3, 6, 9, 12, 15, 18, and 21 days using an alkaline phosphatase assay quantification kit (JianCheng, China). The results were measured spectrophotometrically at a wavelength of 520 nm, based upon the manufacturer's instructions.

### 2.7. Alizarin Red Staining and Quantification

Cells were seeded into 24-well plates at a density of approximately 1 × 10^5^ cells per well separately. After cells reached 80% confluence, the medium was changed into standard osteogenic differentiation induction medium and then cultured for another 3 weeks. The induction medium was changed every 3 days. Finally, cells were stained with Alizarin red (pH = 4.1) staining solution and were quantified according to previously described methods [10].

### 2.8. Oil Red O Staining

Cells were seeded into 24-well plates at a density of approximately 1 × 10^5^ cells per well. After the cells reached 80% confluence, the medium was changed into standard adipogenic differentiation induction medium (0.5 mmol/L of methylisobutylxanthine (Sigma, USA), 1 mmol/L of dexamethasone, 10 *μ*g/mL of insulin (Sigma, USA), and 200 *μ*mol/L of indomethacin (Sigma, USA)) and then cultured for another 3 weeks. The induction medium was changed every 3 days. Finally, cells were stained with Oil red O staining solution and microscopically observed under phase contrast (Zeiss, Germany).

### 2.9. Immunofluorescence

Cells were seeded into confocal dishes at approximately 1 × 10^5^ cells per well. After adherence, the culturing medium was changed into neurogenic differentiation induction medium (Gibco, USA) containing 20 ng/mL of EGF (Calbiochem, USA) and 20 ng/mL of bFGF (Calbiochem, USA) and then cultured for another 2 weeks. The induction medium was changed every 3 days. Finally, cells were stained with mouse anti-human *βIII-TUBULIN* primary antibody (Cell Signaling Technology, USA) at a concentration of 1 : 100. After overnight incubation, the cells were stained with DyLight 405 (ThermoFisher, USA) at a concentration of 1 : 50 and Hoechst dye (RiboBio, China) at a concentration of 1 : 100. Then, the cells were imaged with a laser scanning confocal microscope (Zeiss, Germany).

### 2.10. Quantitative Real-Time Polymerase Chain Reaction (Q-PCR)

Cells were seeded into 60-mm dishes. At 80% confluence, cells were induced by osteogenic/adipogenic/neurogenic differentiation medium for 2 weeks. Then, the total RNA was extracted from cells using Trizol reagent (TAKARA, Japan), and a reverse-transcription reaction was performed using a TAKARA reverse transcriptase kit (TAKARA, Japan). Real-time PCR was performed using a standard SYBR Green PCR kit (TAKARA, Japan) protocol on an Applied Biosystems 7500 real-time PCR system (Applied Biosystems, USA), according to the manufacturer's instructions. *β-ACTIN* was used as an internal reference. The mRNA expression of osteogenic, adipogenic, and neurogenic marker genes was analyzed. Each sample was analyzed in triplicate. The 2^−ΔΔCt^ value was used to determine the relative expression levels. The results were expressed as log_10_⁡(2^−ΔΔCt^). The gene names and primer sequences are shown in Table 2.

### 2.11. Statistical Analysis

Each experiment was performed at least three times, unless otherwise indicated. Data are reported as the mean ± SD (Standard Deviation). The significance of the differences between the experimental and the control groups was determined by using post hoc analysis for ANOVA. A value of *P* < 0.05 was considered to be statistically significant.

## 3. Results

### 3.1. The Characterization and* ANCR* Interference of DTSCs

**Figure 1:**
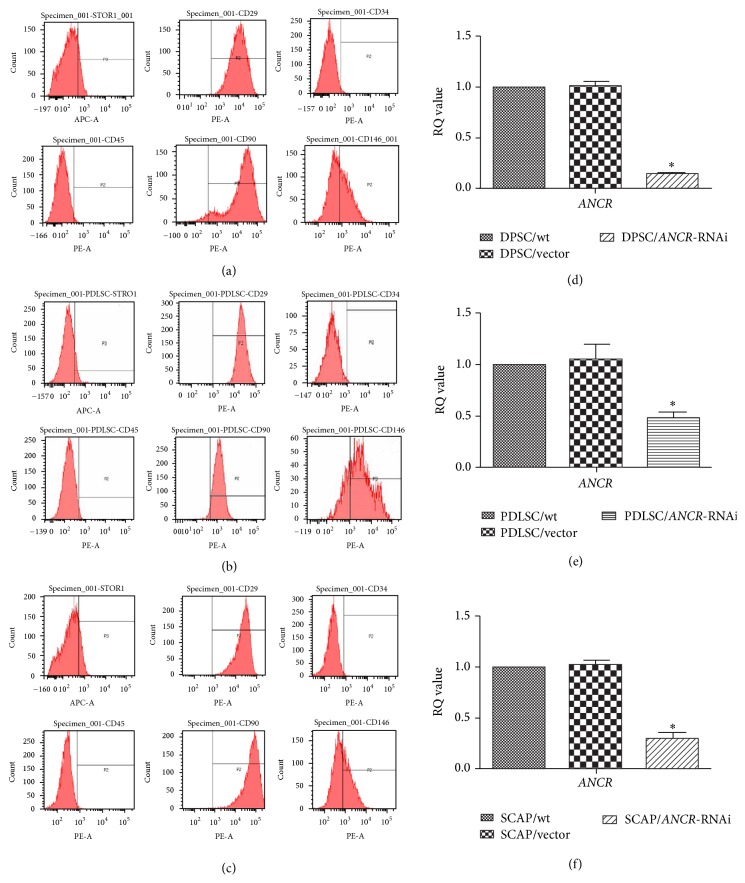
The characterization and* ANCR* interference of DTSCs. The DPSCs (a), PDLSCs (b), and SCAP (c) that we isolated expressed mesenchymal stem cell markers, including* CD29, CD90, CD146,* and* STRO-1*, but were negative for the hematopoietic cell marker* CD34* and leukocyte marker* CD45*. Three days after lentivirus infection, we evaluated the effects of* ANCR*-RNAi by Q-PCR. The results showed that* ANCR* expression in DPSC/*ANCR*-RNAi (d), PDLSC/*ANCR*-RNAi (e), and SCAP/*ANCR*-RNAi (f) cells was remarkably downregulated when compared with control cells. ^*∗*^ *P* < 0.05 when compared with the control group.

Immunophenotype analysis showed that the separated DPSCs (Figure 1(a)), PDLSCs (Figure 1(b)), and SCAP (Figure 1(c)) were* CD29, CD90, CD146,* and* STRO-1* positive and* CD34* and* CD45* negative. Q-PCR analysis was performed 3 days after infection. The results showed that* ANCR* was significantly knocked down by the* ANCR*-specific lentivirus-delivered shRNA in the* ANCR*-RNAi groups compared with control groups (wt and vector) in DPSCs (Figure 1(d)), PDLSCs (Figure 1(e)), and SCAP (Figure 1(f)).

### 3.2. The Effect of* ANCR*-RNAi on DTSC Proliferation

**Figure 2:**
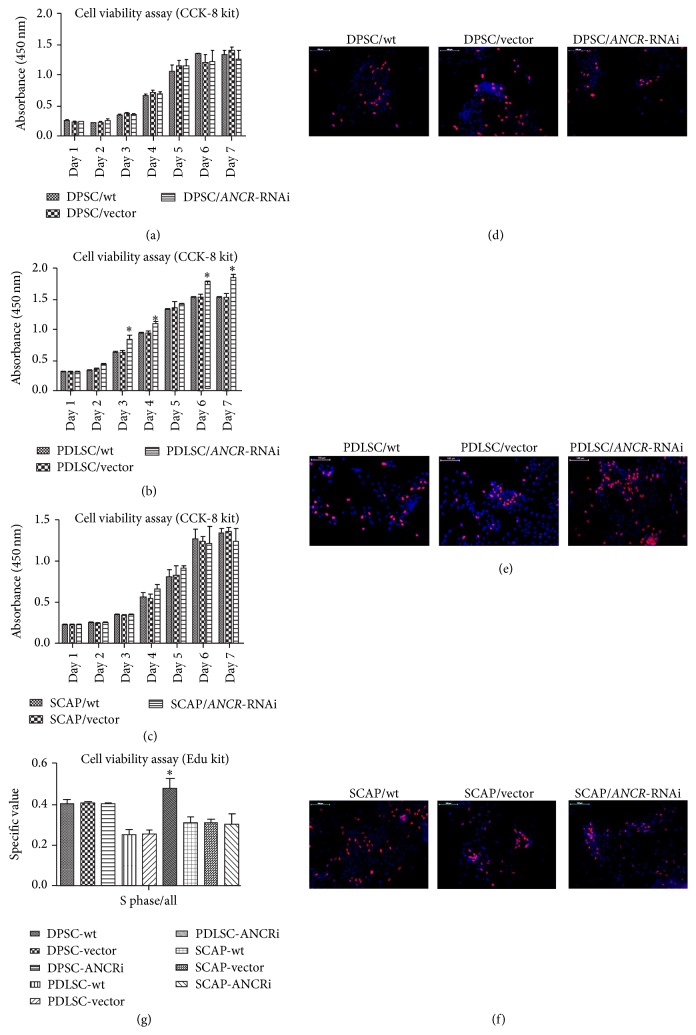
The effects of* ANCR*-RNAi on DTSCs proliferation. From day 1 to day 7, the cell viability of PDLSC/*ANCR*-RNAi cells (b) was greater than that of the control cells; however, there were no statistically significant differences in DPSCs (a) and SCAP (c). Edu assay showed that there were more PDLSC/*ANCR*-RNAi cells (e) in S phase (purple) than control cells. However, there was little difference between* ANCR*-RNAi cells and control cells in DPSCs (d) and SACP (f). The cell counting result confirmed this result (g). The specific value of cells in S phase was higher in PDLSC/*ANCR*-RNAi cells compared to the control group; however, there were no statistically significant differences in DPSCs and SCAP. ^*∗*^ *P* < 0.05 when compared with the control group. Bars 100 *μ*m.

A cell viability assay using CCK8 kit was performed to quantify the proliferation of DTSCs. The results showed that the growth rate of cells from PDLSC/*ANCR*-RNAi group was increased compared with the control groups (Figure 2(b)). However, downregulating* ANCR* had little impact on DPSCs (Figure 2(a)) and SCAP (Figure 2(c)) proliferation. To confirm this result, we used Edu kit to analyze the cell viability. The results showed that there were more PDLSC/ANCR-RNAi cells (Figure 2(e)) in S phase (purple) than control cells. However, there was little difference between* ANCR*-RNAi cells and control cells in DPSCs (Figure 2(d)) and SACP (Figure 2(f)). Cell counting by Image J confirmed this result (Figure 2(g)). In summary, downregulating* ANCR* promoted the cell viability of PDLSCs but had little effect on the cell viability of DPSCs and SCAP.

### 3.3. Downregulation of* ANCR* Promoted the Osteogenic Differentiation of DTSCs

**Figure 3:**
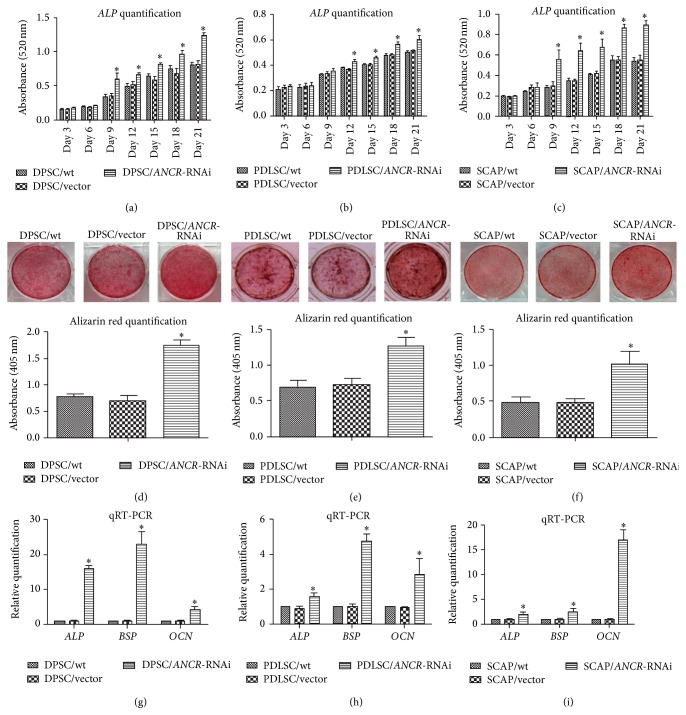
The effect of* ANCR*-RNAi on osteogenic differentiation in DTSCs.* ALP* activity was significantly increased in DPSC/*ANCR*-RNAi (a), PDLSC/*ANCR*-RNAi (b), and SCAP/*ANCR*-RNAi (c) cells when compared with control cells. Three weeks after induction, the cells were stained with Alizarin red (pH = 4.1), and DPSC/*ANCR*-RNAi (d), PDLSC/*ANCR*-RNAi (e), and SCAP/*ANCR*-RNAi (f) cells formed more mineralized nodules than the control groups. The Alizarin red quantification confirmed this result. The mRNA expression of* ALP*,* BSP,* and* OCN* was upregulated in DPSC/*ANCR*-RNAi (g), PDLSC/*ANCR*-RNAi (h), and SCAP/*ANCR*-RNAi (i) cells compared with control cells after two weeks of induction. ^*∗*^ *P* < 0.05 when compared with the control group.

*ALP* activity quantification showed that downregulating* ANCR* increased* ALP* secretion in DTSCs. A significant difference appeared at day 9 in DPSCs (Figure 3(a)) and SCAP (Figure 3(c)) but not until day 12 in PDLSCs (Figure 3(b)). The Alizarin red staining showed that* ANCR*-RNAi cells secreted more mineralizing matrix than the control groups. Quantification showed that the absorbance of DPSC/*ANCR*-RNAi cells (Figure 3(d)) was twofold higher than the control groups and the absorbance of PDLSC/*ANCR*-RNAi (Figure 3(e)) and SCAP/*ANCR*-RNAi (Figure 3(f)) cells was onefold higher than the control groups. Q-PCR showed that all of the osteogenic marker genes (*ALP*,* BSP*, and* OCN*) were upregulated in* ANCR*-RNAi cells (Figures 3(g)–3(I)).

### 3.4. Downregulation of* ANCR* Promoted the Adipogenic Differentiation of DTSCs

**Figure 4:**
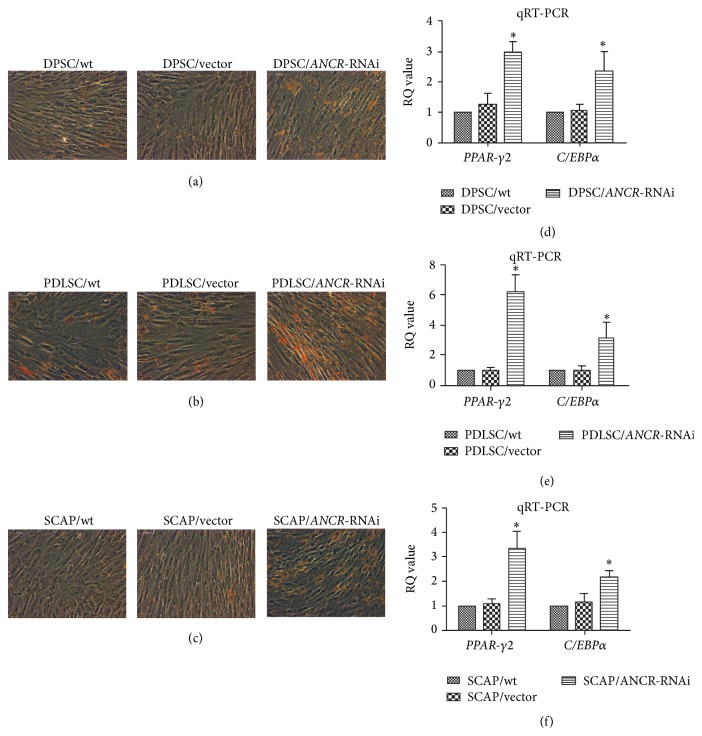
The effect of* ANCR*-RNAi on adipogenic differentiation in DTSCs. Oil red O staining showed that DPSC/*ANCR*-RNAi (a), PDLSC/*ANCR*-RNAi (b), and SCAP/*ANCR*-RNAi (c) cells formed more lipid droplets than the control groups. The mRNA expression of* PPAR-γ2* and* C/EBPα* was upregulated in DPSC/*ANCR*-RNAi (d), PDLSC/*ANCR*-RNAi (e), and SCAP/*ANCR*-RNAi (f) cells compared with control cells after 2 weeks of induction. ^*∗*^ *P* < 0.05 when compared with the control group.

Oil red O staining showed that downregulating* ANCR* promoted adipogenic differentiation of DPSCs (Figure 4(a)), PDLSCs (Figure 4(b)), and SCAP (Figure 4(c)). Q-PCR showed that all of the adipogenic marker genes (*PPARγ-2* and* C/EBPα*) were upregulated in DPSCs (Figure 4(d)), PDLSCs (Figure 4(e)), and SCAP (Figure 4(f)).

### 3.5. Downregulation of* ANCR* Promoted the Neurogenic Differentiation of DTSCs

**Figure 5:**
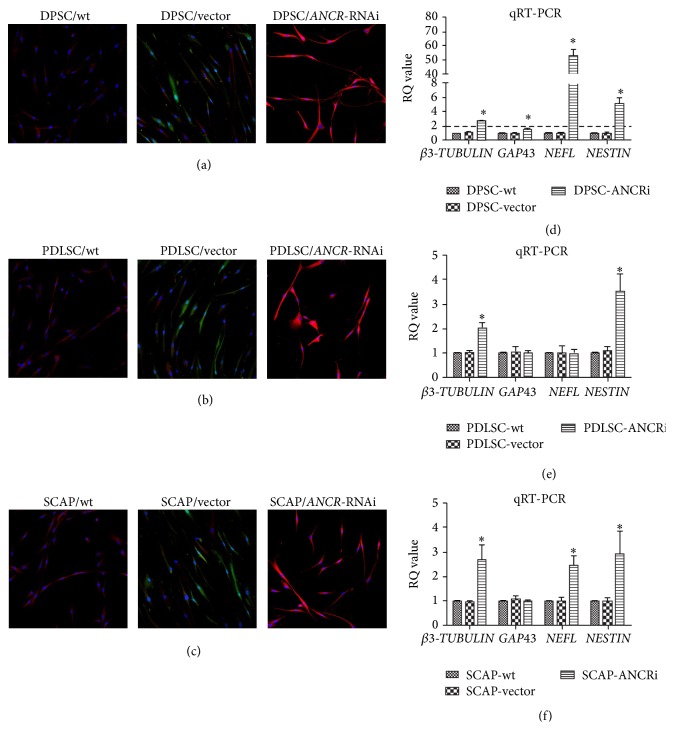
The effect of* ANCR*-RNAi on neurogenic differentiation in DTSCs. Immunofluorescence results showed that the expression of *βIII-TUBULIN* on DPSC/*ANCR*-RNAi (a), PDLSC/*ANCR*-RNAi (b), and SCAP/*ANCR*-RNAi (c) cells was stronger than control groups. The green is the color of GFP (Green Fluorescent Protein) transfected by the vector. The red is the color of *βIII-TUBULIN* stained by DyLight 405. The morphology of DPSC/*ANCR*-RNAi and PDLSC/*ANCR*-RNAi cells was remarkably changed. The mRNA expressions of *βIII-TUBULIN*,* GAP43*,* NEFL,* and* NESTIN* were upregulated in DPSC/*ANCR*-RNAi cells (d) after 2 weeks of induction. In PDLSC/*ANCR*-RNAi cells (e), *βIII-TUBULIN* and* NESTIN* were upregulated, while* GAP43* and* NEFL* had little change compared with control groups. In SCAP/*ANCR*-RNAi cells (f), *βIII-TUBULIN*,* NEFL*, and* NESTIN* were upregulated while* GAP43* had little change compared with control groups. ^*∗*^ *P* < 0.05 when compared with the control group.

Immunofluorescence showed stronger *βIII-TUBULIN* expression in DPSC/*ANCR*-RNAi (Figure 5(a)), PDLSC/*ANCR*-RNAi (Figure 5(b)), and SCAP/*ANCR*-RNAi (Figure 5(c)) cells than in the control cells. After two weeks of induction, the cellular morphology of DPSC/*ANCR*-RNAi and PDLSC/*ANCR*-RNAi cells was markedly changed compared with control groups. DPSC/*ANCR*-RNAi cells were connected by an axon/dendron-like structure that extended from the cell body. The cellular morphology of the PDLSC/*ANCR*-RNAi cells became irregular triangles or oval. However, there was little change in the cellular morphology of SCAP/*ANCR*-RNAi cells. Q-PCR showed that *βIII-TUBULIN*,* GAP43*,* NEFL*, and* NESTIN* were upregulated in DPSC/*ANCR*-RNAi cells (Figure 5(d)) compared with the control groups. In PDLSC/*ANCR*-RNAi cells (Figure 5(e)), *βIII-TUBULIN* and* NESTIN* were upregulated, while* GAP43* and* NEFL* had little change compared with the control groups. In SCAP/*ANCR*-RNAi cells (Figure 5(f)), *βIII-TUBULIN*,* NEFL*, and* NESTIN* were upregulated while* GAP43* had little change compared with the control groups.

## 4. Discussion

Compared with other mesenchymal stem cells (MSCs) derived from bone marrow, adipose tissue, peripheral blood, and umbilical cord blood, MSCs derived from dental tissues have marked advantages of easy access with least invasive procedures without any ethical issues [36]. The ability to undergo self-renewal and multidifferentiation is the most remarkable characteristic of DTSCs, which make them particularly suited for tissue engineering and gene therapy application. Before using DTSCs for clinical therapy, the ability of* in vitro* expansion and differentiation should be taken into account. A range of factors participate in the regulation of DTSCs differentiation and proliferation [37]. We herein used a laterally comparative analysis to investigate the role of long noncoding RNA-*ANCR* in regulating the* ex vivo* expansion and differentiation of three types of DTSCs, including DPSCs, PDLSCs, and SCAP.

Under osteogenic conditions, the* ANCR*-RNAi cells highly mineralized compared with the control groups demonstrated by the quantification of the Alizarin red staining, as well as the* ALP* activity. These results indicate that downregulation of* ANCR* may promote osteogenic differentiation of DTSCs. Thus to further confirm osteogenic differentiation thoroughly, we chose to evaluate the expression of several osteogenic related genes (including* OCN*,* BSP*, and* ALP*).* OCN* is secreted solely by osteoblasts and often used as a marker for the bone formation process [38]. Its gene expression is recognized to increase at a late stage of osteoblastic differentiation [39].* BSP*, a component of mineralized tissues such as bone, dentin, and calcified cartilage, is considered as a specific marker for osteoblasts [40]. The gene expression of* OCN*,* BSP*, and* ALP* was upregulated in the* ANCR*-RNAi cells compared with control groups. Taken together, we speculate that the osteogenic differentiation of DTSCs that is promoted by downregulated* ANCR* is implemented by upregulating* OCN*,* BSP*, and* ALP*.

Downregulation of* ANCR* promoted the adipogenic differentiation of DTSCs demonstrated by Oil red O staining and confirmed by* PPAR-γ2* and* C/EBPα* expression. More lipid droplets were form in DTSCs/*ANCR*-RNAi cells compared with the control groups. And the gene expression of* PPAR-γ2* and* C/EBPα* was upregulated after the downregulation of* ANCR*.* PPARγ-2* is known as a transcription factor in the directed differentiation of preadipocytes that is specifically expressed at the early stage of adipocyte differentiation. It is a key factor in adipogenic differentiation.* C/EBPα* works with* PPARγ-2* to promote adipogenic differentiation [41]. We speculate that promotion of adipogenic differentiation caused by downregulating* ANCR* is implemented by upregulating* PPARγ-2* and* C/EBPα*.

*βIII-TUBULIN* is often identified as the structural protein of neuronal cells. It plays a vital role in the process of neurogenic differentiation [42]. After two weeks of neurogenic differentiation induction, we examined the immunofluorescence staining of *βIII-TUBULIN*. Compared with the control groups, the morphology of DPSC/*ANCR*-RNAi cells and PDLSC/*ANCR*-RNAi cells was obviously changed. The morphology of DPSC/*ANCR*-RNAi cells was similar to neuronal-like cells. There were several axon-like structures extending from the cell body that connected with adjacent cells. The morphology of the PDLSC/*ANCR*-RNAi cells was changed from long fusiform cells into irregular triangles or ovals, with no axon-like structures, while the morphology of the control cells became long and thin. This same long, thin morphology was observed in SCAP/*ANCR*-RNAi cells and the control groups. There was little difference between the SCAP/*ANCR*-RNAi cells and control groups. The red fluorescence of *βIII-TUBULIN* became more obvious after* ANCR* downregulation in DPSCs, PDLSCs, and SCAP.

We used Q-PCR to evaluate the gene expression of *βIII-TUBULIN*,* GAP43*, neurofilament-light polypeptide (*NEFL*), and* NESTIN*. The *βIII-TUBULIN* expression was upregulated after downregulation of* ANCR* in DTSCs. These results could explain the increased red fluorescence of *βIII-TUBULIN* in DTSC/*ANCR*-RNAi cells.* NESTIN*, an intermediate filament protein, is an important surface marker on neural progenitor cells [43]. Q-PCR showed that* NESTIN* expression was upregulated after downregulating* ANCR* in DTSCs.* GAP43* or* neuromodulin* is a neural specific axon membrane protein, which plays a vital role in axonogenesis [44]. The expression of* GAP43* was upregulated in DPSCs but was not significantly different in PDLSCs and SCAP.* NEFL* is a key protein in axon intermediate filament formation that also plays an important role in axonogenesis [21]. After* ANCR* downregulation in DPSCs, the expression of* NEFL* was increased more than 50-fold compared with the control groups, indicating that DPSC/*ANCR*-RNAi cells have very strong axonogenesis. However, the expression of* NEFL* was approximately twofold higher in SCAP/*ANCR*-RNAi but largely unchanged in PDLSCs compared with the control groups. We found that the changes in the expression of* GAP43* and* NEFL* were in accordance with the morphology changes of DTSCs/*ANCR*-RNAi cells. From these data, we concluded that downregulating* ANCR* promoted neurogenic differentiation of DTSCs and that the neurogenic differentiation potential of DPSCs was more obviously enhanced by downregulated* ANCR* compared with PDLSCs and SCAP.

As for the proliferation aspect, the regulation effect of* ANCR* on three types of DTSCs was different. Downregulation of* ANCR* promoted the proliferation of PDLSCs but had little effect on DPSCs and SCAP proliferation. We used cck-8 and Edu assay to confirm this results. Previous studies demonstrate that stem cells derived from dental tissues show a difference in terms of cell proliferation [3, 45]. Although most of stem cells from dental tissues exhibit the similar fibroblast-like morphology and clonogenic abilities after* in vitro* expansion, they orient from different niches of postnatal stem cells. The stem cells niches refer to an* in vivo* or* in vitro* stem cells microenvironment which maintain stem cells in a quiescent state [46, 47]. However, following tissue injury, the surrounding microenvironment interacts with stem cells to regulate cell fate. Stem cells behaviours (self-renewal or differentiation for tissue regeneration) are mainly governed by these distinct microenvironment which includes growth factors, cytokines, and extracellular matrix components, as well as cell signaling pathways [48]. A recent study in rats demonstrates that Notch signaling pathway plays a vital role in controlling DTSCs fate during the tooth development [49]. Additionally, our previous research found that* ANCR* promotes the proliferation and osteogenic differentiation of PDLSCs by activating the canonical Wnt signaling pathway [34]. The canonical Wnt signaling pathway which is an ancient and evolutionarily conserved pathway extensively participates in osteogenic, adipogenic, and neurogenic differentiation of stem cells [50]. It has been demonstrated that downregulating* ANCR* promotes osteoblast differentiation by targeting* EZH2* and regulating* Runx2* expression [33].* EZH2* is known to be a key cellular homeostasis and differentiation regulator which interacts with canonical Wnt signaling pathway in stem cells [51]. Based on these researches, we speculate that* ANCR* may interact with* EZH2* via canonical Wnt signaling pathway, thus regulating the DTSCs differentiation. Belonging to lncRNA,* ANCR* acts as the upstream of the regulatory network. It is a novel factor in regulating stem cells behaviours. Therefore, further in-depth investigation shall be performed to fully evaluate the control mechanism of lncRNA-*ANCR* during the proliferation and differentiation of DTSCs, as well as crosstalk between multiple pathways regulated by* ANCR*.

## 5. Conclusion

This study demonstrates that lncRNA-*ANCR* acts as a key regulator in terms of DTSCs proliferation and multiple-potential of differentiation. Downregulation of* ANCR* promoted the proliferation of PDLSCs, as well as osteogenic, adipogenic, and neurogenic differentiation of DTSCs (DPSCs, PDLSCs, and SCAP). More work is required to investigate the related genes and signaling pathways via regulation of* ANCR* controlling the DTSCs behaviours. This study represents a step forward in providing a novel regulator factor for further research in better understanding the behaviours of stem cells from dental tissues.

## Figures and Tables

**Table 1:** The effects of different regulating factors on DTSC.

Research team	Cell	Regulating factors	Regulatory effect
Osathanon et al. [10]	DPSC	BFGF	Inhibit osteogenic differentiation
Dissanayaka et al. [7]	DPSC	VEGF	Promote angiopoietic differentiation
Feng et al. [11]	DPSC	IGF	Promote osteogenic differentiation
He et al. [12]	DPSC	LPS	Promote osteogenic differentiation
Yang et al. [13]	DPSC	IL-1, TNF-*α*	Promote osteogenic differentiation
Gay et al. [14]	DPSC	MicroRNA-218	Inhibit osteogenic differentiation
Hara et al. [15]	DPSC	MicroRNA-720	Promote osteogenic differentiation
Wei et al. [16]	PDLSC	MicroRNA-21	Promote osteogenic differentiation
Ye et al. [17]	PDLSC	BMP9	Promote osteogenic differentiation
Osathanon et al. [18]	PDLSC	BFGF	Inhibit osteogenic differentiation
Chen et al. [19]	PDLSC	NF*κ*-B	Promote osteogenic differentiation
Song et al. [20]	PDLSC	BMP2	Promote adipogenic differentiation
Osathanon et al. [21]	PDLSC	Notch signaling	Promote neurogenic differentiation
Wu et al. [22]	SCAP	BFGF	Inhibit osteogenic differentiation
Wang et al. [23]	SCAP	Canonical Wnt pathway	Promote osteogenic differentiation
Zhang et al. [24]	SCAP	BMP2	Promote osteogenic differentiation
Li et al. [25]	SCAP	17*β* estradiol	Promote osteogenic differentiation

**Table 2:** Primer sequences.

	Primer	Sequence (5′-3′)
	*β-ACTIN*-forward	TGGCACCCAGCACAATGAA
	*β-ACTIN*-reverse	CTAAGTCATAGTCCGCCTAGAAGCA
	*ANCR*-forward	GCCACTATGTAGCGGGTTTC
	*ANCR*-reverse	ACCTGCGCTAAGAACTGAGG

Osteogenic differentiation marker genes	*ALP*-forward	CCACGTCTTCACATTTGGTG
	*ALP*-reverse	AGACTGCGCCTGGTAGTTGT
	*OCN*-forward	GGCAGCGAGGTAGTGAAGAG
	*OCN*-reverse	CTGGAGAGGAGCAGAACTGG
	*BSP*-forward	AAAGTGAGAACGGGGAACCT
	*BSP*-reverse	GATGCAAAGCCAGAATGGAT

Adipogenic differentiation marker genes	*PPAR-γ2*-forward	CATTCTGGCCCACCAACTT
	*PPAR-γ2*-reverse	CCTTGCATCCTTCACAAGCA
	*C/EBPα*-forward	TGGACAAGAACAGCAACGAG
	*C/EBPα*-reverse	TTGTCACTGGTCAGCTCCAG

Neurogenic differentiation marker genes	*βIII-TUBULIN*-forward	GGCCTCTTCTCACAAGTACG
	*βIII-TUBULIN*-reverse	CCACTCTGACCAAAGATGAAA
	*GAP43*-forward	CCTGTGGGAGTCCACTTTCC
	*GAP43*-reverse	CGATATTTTGGACTCCTCAGATGA
	*NEFL*-forward	TGATGGTAATGGATTGGAACTATGA
	*NEFL*-reverse	TCAACCCAGGTCTAGTAAGCAGAA
	*NESTIN*-forward	GTAGCTCCCAGAGAGGGGAA
	*NESTIN*-reverse	CTCTAGAGGGCCAGGGACTT
